# Master protocol to assess the long-term safety in kidney transplant recipients who previously received Medeor’s cellular immunotherapy products: the MDR-105-SAE

**DOI:** 10.1186/s13063-023-07204-4

**Published:** 2023-03-11

**Authors:** Sam Kant, Dixon B. Kaufman, Lenuta Micsa, Daniel C. Brennan

**Affiliations:** 1 Department of Medicine, Johns Hopkins University School of Medicine, Baltimore, MD USA; 2 Department of Surgery, School of Medicine and Public Health, University of Wisconsin-Madison, Madison, WI USA; 3 Medeor Therapeutics, Inc., 611 Gateway Blvd., Suite 120, South San Francisco, CA 94080 USA

**Keywords:** Tolerance, Kidney transplantation, Immunotherapy, Immunosuppression

## Abstract

**Background:**

Immunosuppression in transplantation continues to be associated with a multitude of adverse effects. Induction of immune tolerance may be a viable strategy to reduce dependence on immunosuppression. Various trials are currently underway to assess the efficacy of this strategy. However, long-term safety data for these immune tolerance regimes has yet to be established.

**Methods/design:**

At the completion of primary follow-up of various Medeor kidney transplant studies, subjects receiving cellular immunotherapy products will be followed annually as per protocolized schedule for up to an additional 84 months (7 years) to evaluate long-term safety. Long-term safety will be assessed by summarizing incidence of serious adverse events, adverse events leading to study withdrawal and hospitalization rates.

**Discussion:**

This extension study will be an important step in evaluating safety issues pertaining to immune tolerance regimens, long-term effects of which are largely unknown. These data are essential for furthering an unrealized goal of kidney transplantation- graft longevity without the adverse effects from long-term immunosuppression. The study design utilizes the methodology of a master protocol, wherein multiple therapies can be assessed simultaneously with accompanied gathering of long-term safety data.

## Background

Thefield of transplantation is actively engaged in studying the induction of effective immune tolerance without the use of immunosuppression. Clinical trials to establish tolerance are using a combination of organ and hematopoietic cell transplantation to achieve immunological tolerance via either full or mixed donor chimerism [1–3]. Full donor chimerism is acquired when the entire recipient hematopoietic system is replaced by donor cells (donor cells > 98%) resulting in deactivation of donor T cells [4]. Mixed chimerism is defined as continued mixing of donor and recipient hematopoietic cells in recipient tissues after transplantation of donor cells. Achievement of mixed chimerism can result in tolerance of organ grafts from the hematopoietic cell donors without the need for immunosuppression [3]. Various centers have employed myeloablative or non-myeloablative conditioning strategies, with total body, local thymic, or lymphoid irradiation, and subsequent donor whole bone marrow transplantation or infusion of granulocyte colony stimulating factor (G-CSF) mobilized CD34 + T cells [5–7]. Establishment of mixed chimerism is associated with lower risk of graft versus host disease (GVHD) and immunodeficiency in comparison to full donor chimerism. The Stanford group reported the long-term outcomes of a pioneering, single-center study in which kidney transplant recipients were given post-transplant conditioning with 10 doses of total lymphoid irradiation (TLI) and 5 doses of anti-thymocyte globulin (ATG) [3]. On completion of TLI, a combination of enriched CD34 + hematopoietic progenitors and a defined number of donor T cells were injected immediately (G-CSF was administered to donors to mobilize progenitors into the blood such that collection was performed via apheresis). Fully HLA-matched patients were given T cells along with the CD34 + cells. The majority of the patients (24/29 fully HLA matched) achieved mixed chimerism for at least 1 year. In the mixed chimerism group, MMF was withdrawn 39 days after cell infusion following kidney transplantation, while calcineurin inhibitors were tapered beginning at approximately 6 months and ceased after approximately 1 year. In the Stanford study, 23/24 (96%) had no evidence of rejection in the first year after withdrawal of immunosuppression. Although two late rejections occurred at 44 and 60 months after rejection, no grafts were lost due to rejection [8]. For this strategy to achieve success, it is imperative to elucidate the long-term safety profile. The monitoring of events such as rejection, graft dysfunction/loss, graft vs host disease (GVHD), and opportunistic infections is important. The MDR-105-SAE study is designed to be a long-term safety monitoring extension trial involving Medeor’s cellular immunotherapy administered to kidney transplant recipients to induce immune tolerance via mixed lymphohematopoietic chimerism. MDR-105-SAE is a Master Protocol that is designed to allow current and future kidney transplant studies to enroll into a single design where safety data can be analyzed in a consistent manner. The initial clinical trial to be enrolled is a phase 3 open label, multicenter, randomized controlled trial to assess safety and efficacy of donor-derived CD34 + hematopoietic stem/progenitor cells and a specified dose of CD3 + T cells administered to recipients of HLA-matched, living donor kidney transplants post total lymphoid irradiation (TLI) and rabbit-anti-thymoglobulin induction, with progressive tapering of maintenance immunosuppression (NCT03363945).

This study protocol will provide up to 84 months (7 years) of additional follow-up to collect the data into a centralized database allowing for continuous monitoring of any important safety signals.

## Methods

### Patient population

The study population will consist of kidney transplant recipients who received Medeor’s cellular immunotherapy in its previous and future kidney transplant studies. Upon completion of the initial study, the kidney transplant recipients who received the Medeor product are given an option to participate in the long-term follow-up study, MDR-105-SAE. Study participants with subsequently failed allografts, albeit now on kidney replacement therapy, will also be included in this study. The study will exclude subjects who have lost kidney allografts and have been subsequently retransplanted.

#### Subject inclusion criteria

All of the following criteria must be met for study participants to be included in the study:

1. Able and willing to fully comply with all study procedures and restrictions.
2. Able to understand and provide written, signed, and dated informed consent to participate in the study in accordance with International Council for Harmonization (ICH) Good Clinical Practice Guidelines (GCP) and all applicable local regulations.
3. Have previously completed a Medeor study and received a Medeor cellular immunotherapy product

#### Subject exclusion criteria

Participants who meet any of the following criteria will not be eligible:

1. Has any condition or circumstance, which in the opinion of the investigator would significantly interfere with the subject’s protocol compliance or put the subject at increased risk. The subject will be expected to comply with all study visits and procedures. The protocol requires annual study visits where a complete physical exam, vital signs, and laboratory samples will be obtained.
2. Unable or unwilling to provide written, signed, and dated informed consent to participate in the study.
3. Has undergone a second organ transplant with an organ derived from an individual other than the donor of the transplant kidney received during a Medeor study

### Baseline characteristics

Participant demographics, past medical history, and clinical course will be recorded. To be able to link the subject data from this study to the parent study, the study code, center number, subject number, and the last visit date from the parent study will be recorded in the case report form (CRF).

### Study design

At the time of enrolment in the initial study, the kidney transplant recipients are asked to acknowledge the expectation to participate in a multi-year, long-term follow-up study at the completion of the observation period in the initial study. Upon completion of the initial study, the kidney transplant recipients who received the Medeor product will sign the consent form and begin Study MDR-105-SAE. These patients are expected to participate in the MDR-105-SAE study. Medeor is working with the sites to be able to enroll these subjects into the MDR-105-SAE study upon completion of the initial study. At the completion of primary follow-up of various Medeor kidney transplant studies (called parent studies), subjects receiving Medeor’s cellular immunotherapy products will be followed annually for up to an additional 84 months (7 years) for long-term safety evaluation. No study treatment is involved. All immunosuppression and therapeutic drug monitoring will be managed by treating physicians according to local standard of care (SOC). Any changes in, and reinstitution of immunosuppression drug therapy, will be at the treating physician’s discretion. Post-kidney transplant biopsy will be performed for cause at any time at the discretion of a subject’s treating physician. MDR-105-SAE protocol assessments will be performed annually following study entry. All participants must provide informed consent prior to enrolling into the MDR-105-SAE study.

### Study visits and procedures

Within 3 months of completion of a Medeor clinical trial, the kidney transplant recipients who received a Medeor cellular immunotherapy product will begin annual study MDR-105-SAE visits at 12 months (with an allowed deviation of ± 1 month for scheduled annual visits).

Unscheduled study visits at the transplant center and at other health care delivery facilities may occur. Records from such visits would be obtained and reviewed for adequate safety reporting.

Extensions in study visit scheduling to accommodate non-working days will not be considered protocol deviations. Additionally, extensions in scheduling because of special circumstances, such as subject work issues, lack of transportation, and bad weather, if cleared in advance with the medical monitor, will also not be considered protocol deviations.

The schedule for clinical assessments is elucidated in Table 1 (below references).

**Table 1:** Schedule of monitoring over the course of MDR-105-SAE

**Study visit year**	1		2	3	4	5	6	7
**Study visit number**	0	1	2	3	4	5	6	7
**Study visit window (months)**	+ 3	± 1	± 1	± 1	± 1	± 1	± 1	± 1
**Assessment**	**Screening**	**Post enrollment**						
Informed consent	⦁							
Confirm study eligibility criteria	⦁							
Demographics	⦁							
Baseline characteristics	⦁							
Medical history	⦁							
Prior/concomitant medication	⦁	⦁	⦁	⦁	⦁	⦁	⦁	⦁
Perform complete physical examination	⦁	⦁	⦁	⦁	⦁	⦁	⦁	⦁
Record vital signs	⦁	⦁	⦁	⦁	⦁	⦁	⦁	⦁
Record weight	⦁	⦁	⦁	⦁	⦁	⦁	⦁	⦁
Record height	⦁							
Hematology panel^a^		L	L	L	L	L	L	L
Comprehensive metabolic panel^a^		L	L	L	L	L	L	L
Urinalysis		L	L	L	L	L	L	L
Calcineurin inhibitor trough levels^b^		L	L	L	L	L	L	L
Mixed chimerism testing^c^		C	C	C	C	C	C	C
Record instances of acute rejection requiring treatment		⦁	⦁	⦁	⦁	⦁	⦁	⦁
Record instances of proteinuria		⦁	⦁	⦁	⦁	⦁	⦁	⦁
Kidney biopsy^d^		Only for cause						
Complete subject status form		⦁	⦁	⦁	⦁	⦁	⦁	⦁
Record all SAEs		⦁ (ongoing)						
Record AEs leading to study withdrawal		⦁ (as needed)						
Record history of hospitalizations since transplant		⦁	⦁	⦁	⦁	⦁	⦁	⦁
Record development of NODAT^e^		⦁	⦁	⦁	⦁	⦁	⦁	⦁
Record development of GVHD		⦁	⦁	⦁	⦁	⦁	⦁	⦁
Record instances of BK viremia development		⦁	⦁	⦁	⦁	⦁	⦁	⦁
Record instances of dnDSA		⦁	⦁	⦁	⦁	⦁	⦁	⦁
Record instances of cardiovascular events^f^		⦁	⦁	⦁	⦁	⦁	⦁	⦁
Record instances of opportunistic infection		⦁	⦁	⦁	⦁	⦁	⦁	⦁
Record instances of PTLD/other malignancies		⦁	⦁	⦁	⦁	⦁	⦁	⦁
Record instances of MDS		⦁	⦁	⦁	⦁	⦁	⦁	⦁

*AE* adverse effects, *C* central laboratory, *dnDSA* de novo DSA, *L* local laboratory, *MDS* myelodysplastic syndrome, *NODAT* new-onset diabetes after transplantation, *PTLD* post-transplant lymphoproliferative disease

^a^Testing will be completed at each follow-up visit and will be analyzed locally using accepted laboratory methodology

^b^For subjects who have discontinued CNI, compliance check is performed as needed to assess trough levels locally, per standard accepted laboratory methodology. CNI trough levels will be assessed in all recipients at each follow-up visit for subjects taking CNI

^c^The degree of donor chimerism in recipient blood will be by central laboratory. Subjects who have lost mixed chimerism (< 5%) do not need to repeat testing at subsequent visits

^d^Transplant kidney biopsy will be performed for cause at the discretion of a subject’s treating physician and will undergo local pathology review

^e^NODAT will be determined based on need for use of an antidiabetic agent for more than 30 days, or 2 fasting plasma glucose levels ≥ 126 mg/dL in a subject who was not diabetic at study entry

^f^Cardiovascular events include acute myocardial infarction, stroke, acute peripheral arterial occlusion, and revascularization procedure

### Treatments

There will be no protocol mandated treatments or interventions outside of routine post-transplant monitoring as annotated in Table 1. Any interventions, alterations, and/or reinstitution of immunosuppression will be at the sole discretion of the treating physician. Reinstitution of transplant immunosuppression in subjects who successfully achieved functional immune tolerance as defined in the parent study should only be for cause (e.g., biopsy-proven acute rejection). Should immunosuppression be reinstituted, the subject should continue in the study and follow the schedule of assessment given in Table 1. The only non-SOC monitoring will be assessment of mixed chimerism on an annual basis in subjects who maintain the mixed donor chimerism (> 5%). As this is a strictly observational study, there is no rationale for early termination rules or criteria.

### Discontinuation and withdrawal

1. Withdrawal of individual subject(s)

A subject is free to withdraw from the study at any time without giving reason for doing so and without penalty or prejudice. The investigator is also free to terminate a subject’s involvement in the study at any time if a subject’s clinical condition warrants it.

Subjects who withdraw all consent for further participation in the study will be withdrawn from all follow-up assessments and will only be followed for mortality through public records. Subjects should have an end of the study visit completed shown in Table 1 at the time of their discontinuation.

(2) Discontinuation of the site

If an investigator intends to discontinue participation in the study, the investigator must.immediately inform the sponsor. The sponsor will provide written notice for sitetermination.

(3) Discontinuation of the study

The sponsor may terminate this study prematurely, either in its entirety or at any study site, for reasonable cause provided that written notice is submitted in advance of the intended termination. If the sponsor terminates the study for noncompliance reasons, the sponsor will immediately notify the investigators and subsequently provide written instructions for study termination.

### Assessment of safety

Safety will be assessed using incidence of serious adverse effects (SAEs) and adverse effects (AEs) leading to study withdrawal (SAEs and AEs defined below); review of laboratory data, including hematology, biochemistry (comprehensive metabolic profile), and urinalysis; hospitalization rates; and vital signs yearly during the study follow-up period.

The coding dictionary for this study will be the Medical Dictionary for Regulatory Activities (MedDRA). It will be used to summarize AEs by system organ class and/or preferred term. The version of MedDRA to be used will be determined prior to final database lock.

### Safety parameters

- Vital signs

All recipients will have vital signs (systolic and diastolic blood pressure, pulse, respiratory rate, and temperature) collected at all yearly study visits. After the subject has been sitting for 5 min, with back supported and both feet placed on the floor, systolic and diastolic blood pressure will be measured with an appropriately sized cuff and calibrated machine.

(b) Physical examination

A complete physical examination will be performed on all recipient subjects as part of the annual safety evaluation study visits. Significant findings that are present prior to signing the Informed Consent Form must be included in the Medical History screen on the subject’s case report form (CRF). The investigator or a qualified designee will conduct the exams, determine findings, and assess any abnormalities as to clinical significance.

(c) Laboratory assessments

Clinical laboratory tests, including hematology and comprehensive metabolic panel (chemistry), will be performed on recipients as specified in the Schedule of Assessments and Procedures in Table 1. Clinical laboratory tests are to be performed and reviewed by the investigator or qualified designee:

- Hematology: complete blood count (CBC) with differential will be measured locally for recipients annually.
- Blood chemistry (Comprehensive Metabolic Panel): A Comprehensive Metabolic Profile must include all of the following measurements: blood urea nitrogen (BUN), creatinine, sodium, potassium, chloride, bicarbonate, glucose, phosphorus, total protein, albumin, creatine kinase (CK), lactate dehydrogenase (LDH), amylase, and liver chemistries including total and direct bilirubin, alanine aminotransferase (ALT), aspartate aminotransferase (AST), and alkaline phosphatase (ALP). The full chemistry panel will be analyzed locally using accepted laboratory methodology at each of the yearly visits.
- Urinalysis: Instances of proteinuria and/or microscopic hematuria will be recorded.

### Safety monitoring

#### Definition of adverse event (AE)

An AE is any unfavorable and unintended diagnosis, symptom, sign (including an abnormal laboratory finding), syndrome or disease which either occurs during the study, having been absent at baseline, or, if present at baseline, appears to worsen.

#### Definition of serious adverse event (SAE)

An SAE is defined (21CFR 312.32) as any adverse event that results in any of the following outcomes:

- Death
- A life-threatening adverse event
- Inpatient hospitalization or prolongation of existing hospitalization
- Persistent or significant incapacity or substantial disruption of the ability to conduct normal life functions
- A congenital anomaly or birth defect

Other important medical events may also be considered an SAE when, based upon appropriate medical judgment, they may jeopardize the patient or subject and may require medical or surgical intervention to prevent one of the outcomes listed above.

### Events of special interest

Clinically significant opportunistic infections (grade 3 Common Terminology Criteria for Adverse Events (CTCAE) or higher or meeting criteria of an SAE), all cancers other than PTLD, and cardiovascular events including revascularization procedures will be reported on dedicated electronic case report forms (eCRFs) for these events of special interest.

- Active surveillance for myelodysplastic syndrome (MDS)

Myelodysplastic syndromes (MDS) include a group of clonal myeloid neoplasms with presence of cytopenias due to ineffective hematopoiesis, abnormal blood, and marrow cell morphology [9]. These abnormal cells can undergo changes in clonality and progress to acute myeloid leukemia (AML). Some myelodysplastic syndromes have no known cause. Others are caused by exposure to cancer treatments, such as chemotherapy and radiation, or to toxic chemicals, such as tobacco, benzene, and pesticides, or to heavy metals, such as lead.

Active surveillance for MDS consists of full annual physical exam with medical history and evaluation for possible risk factors and CBC with platelets and differential. Additional tests such as vitamin B12 and folate may be required when results of CBC and physical exam are outside of normal parameters which often signal an underlying medical issue. MDS will not be reported as AEs, but as outcomes, and will be collected and reported on dedicated case report forms specifically designed to capture these events.

(b) Reporting of pregnancy

Pregnancy is neither an AE nor an SAE, unless a complication relating to the pregnancy occurs. All reports of congenital abnormalities/birth defects are SAEs. Spontaneous miscarriages should also be reported and addressed as SAEs. Elective abortions without complications will not be considered AEs. However, all pregnancies occurring during this study (in subjects or female partners of subjects) are to be reported to the sponsor at the subsequent scheduled visit.

(c) Events to be reported as outcomes rather than adverse events

Rejection episodes, opportunistic infections, BK viremia, cardiovascular events, graft versus host disease (GvHD), post-transplant lymphoproliferative disorder (PTLD), MDS, and new-onset diabetes after transplantation, graft loss, and death are outcomes and will be reported on dedicated case report forms (CRFs) for these events. Causes of death should be reported as SAEs.

(iv) Cardiovascular events

Cardiovascular events will include documented acute myocardial infarction, stroke, acute peripheral arterial occlusion, and revascularization procedures. Revascularization procedures will include open and endovascular procedures intended to restore arterial blood flow. Such procedures include bypass, endarterectomy, thrombectomy, angioplasty, stenting, atherectomy, and aneurysm resection/exclusion. Cardiovascular events will be recorded during the study.

(e) New-onset diabetes after transplantation (NODAT)

NODAT is defined in this study as the use of an antidiabetic agent/insulin for more than 30 days or any of the following in a subject who was not diabetic at study entry [10]:

- Fasting glucose 126 mg/dL (7 mmol/L) in more than one occasion
- Random glucose 200 mg/dL (11.1 mmol/L) with symptoms
- Two-hour glucose after a 75-g oral glucose tolerance test (OGTT) 200 mg/dL (11.1 mmol/L)
- Hemoglobin A1C (HbA1c) 6.5%.

The development of NODAT will be assessed yearly during the study.

(f) Transplant kidney biopsy

Transplant kidney biopsy may be performed for cause at the discretion of a subject’s treating physicians. Biopsies will undergo local pathology review.

(g) Estimated glomerular filtration rate (eGFR)

Th eGFR will be calculated yearly during the study using the 4-variable equation from the CKD-EPI 2021 equation, using locally assessed serum creatinine data from the appropriate time points. The calculation will be done by Data Management based on the laboratory and demographic information collected during the course of the study.

CKD-EPI equation:

$$\mathrm{eGFRcr}=142\times\min\;(\mathrm{Scr}/\kappa,1)\mathrm\alpha\times\max\;(\mathrm{Scr}/\kappa,1)-1.200\times0.9938\mathrm{Age}\times1.012\;\lbrack\mathrm{if}\;\mathrm{female}\rbrack$$

where:

Scr = serum creatinine in mg/dL.

*κ* = 0.7 (females) or 0.9 (males)

*α* =  − 0.241 (female) or − 0.302 (male)

min (Scr/*κ*, 1) is the minimum of Scr/*κ* or 1.0

max (Scr/*κ*, 1) is the maximum of Scr/*κ* or 1.0

Age (years).

(h) De novo DSA (DnDSA)

Post-kidney transplant testing for dnDSA will be performed using single-antigen bead methodology using a Luminex or similar analytic instrument in a central laboratory. The dnDSA testing may also be performed locally at the time of any “for cause” transplant kidney biopsy per institutional standard of care.

(i) Donor chimerism

Mixed chimerism is defined as presence of at least 5% donor cells in either whole blood or in at least one WBC lineage (CD3 + T cells, CD33 + myeloid cells, CD19 + B cells, and/or CD56 + natural killer [NK] cells). The degree of donor chimerism in transplant kidney recipient blood will be assessed centrally using accepted laboratory methodology yearly during the study. Subjects who have lost mixed chimerism (< 5%) do not need to repeat testing at subsequent visits.

### Severity of adverse events

Adverse events, including abnormal clinical laboratory values, will be graded using the National Cancer Institute—Common Terminology Criteria for Adverse Events (NCI-CTCAE) guidelines (Version 5 or higher). Events that are not stipulated in the NCI-CTCAE Version 5 or higher will be assessed according to the criteria below and entered into the eCRF.

It is important to distinguish between serious and severe AEs. An AE of severe intensity may or may not also be considered serious.

### Monitoring and reporting serious adverse events only

All subjects will be monitored closely for SAEs during study participation. Subjects who discontinue prematurely from the study will be encouraged to return for assessment of safety. Follow-up safety information may also be obtained directly from a subject’s physician with source document support whenever applicable (e.g., biopsy results) or from medical records, laboratory reports, and imaging reports.

Whenever possible, symptoms should be grouped as a single syndrome or diagnosis. The investigator should specify the duration (start and end dates) or if the event is ongoing, the severity grade, other medication or therapies have been taken (concomitant medication/non-drug therapy), outcome, and his/her opinion as to whether there is a reasonable possibility that the SAE was caused by the previous study treatment.

In the event of an SAE, the investigator should follow up with the outcome until the clinical recovery is complete and laboratory results have returned to normal or until progression has been stabilized.

The investigator must provide the sponsor appropriate information concerning any findings suggesting significant hazards, contraindications, side effects, or precautions pertinent to the safety of the study treatment. Direct contact information for the Study Medical Monitor will be provided to the institution at time of institutional review.

All SAEs must be reported to the Sponsor within 24 h of the first awareness of the event. The Investigator must complete, sign and date the SAE pages, verify the accuracy of the information recorded on the SAE pages with the corresponding source documents, and send to sponsor:

- The SAEs will be reported to the sponsor by completing an SAE Report Form. All sections on the form are to be completed. Information that is not available at the time of initial reporting should be submitted as a follow-up. The date of receipt of an initial SAE Report Form will be considered as day 0 for the purpose of determining the time for regulatory reporting of an expedited event and timelines of further study-related activities.
- The copy of all examinations carried out and the dates on which these examinations were performed will be attached. For laboratory results, the laboratory normal ranges will be included.

It is the Principal Investigator (PI)’s responsibility to notify the IRB of all SAEs that occur at his or her site. The sponsor is responsible for notifying the relevant regulatory authorities of certain safety events, including expedited safety reports. Investigators will also be notified by the Sponsor of all suspected, unexpected, serious, adverse reactions (SUSAR, 7/15 Day Safety Reports) that occur during the clinical trial. Each site is then responsible for notifying its IRB of these additional SAEs.

### Statistics

#### Safety endpoints

Long-term safety will be assessed by summarizing incidence of serious adverse events (SAEs), AEs leading to study withdrawal, and hospitalization rates. Summary statistics will be presented by visit for laboratory data, including hematology, renal function, biochemistry, and vital signs. Adverse events will be classified for seriousness using standard regulatory criteria and for severity according to the National Cancer Institute—Common Terminology Criteria for Adverse Events (NCI-CTCAE) version 5 or higher grading scale whenever feasible.

#### Outcomes of special interest

The number and proportion of subjects with biopsy-proven acute rejection (BPAR), de novo donor-specific antibody (dnDSA) (if applicable), BK viremia, opportunistic infection, post-transplant lymphoproliferative disorder (PTLD), myelodysplastic syndrome (MDS), new-onset diabetes after transplantation (NODAT), graft versus host disease (GvHD), cardiovascular (CV) events, and initiation of immunosuppression will be presented by visit and overall. Transplant kidney loss and subject death will also be summarized by visit and overall.

Outcomes of special interest noted above will not be reported as AEs, but rather as outcomes, and will be collected and reported on dedicated case report forms specifically designed to capture these events; this will include severity and seriousness assessments.

### Study monitoring

Monitoring and auditing procedures approved by the sponsor will be followed, to comply with GCP guidelines. The study will be monitored by the sponsor or its designee. Monitoring may be done either in person or remotely.

The site monitor will ensure that the investigation is conducted according to protocol design and regulatory requirements by frequent communications (letter, e-mail, telephone).

Regulatory authorities, the IRB, and other appropriate institutional regulatory bodies, and/or the sponsor’s clinical quality assurance group may request access to all source documents, CRFs, and other study documentation for on-site audit or inspection. Direct access to these documents must be guaranteed by the Investigator, who must provide support at all times for these activities.

To ensure compliance with GCP and all applicable regulatory requirements, the sponsor or its designee may conduct a quality assurance audit.

### Ethics

#### Ethics review

For the MDR-105-SAE study, a Central IRB has been approved. Some sites are using a Central IRB while others are using the local institutional review board (IRB). For sites that are using local IRB, the IRB approval must be obtained and the written approval to be submitted to the sponsor or its designee before the site can enroll any subject into the study. The final study protocol, including the final version of the Informed Consent Form, must be approved, or given a favorable opinion in writing by an IRB.

The investigator is responsible for informing the IRB of any amendment to the protocol in accordance with local requirements. The protocol must be re-approved by the IRB upon receipt of amendments and annually if local regulations require.

Initial IRB approval, and all materials approved by the IRB for this study including the subject consent form and recruitment materials must be maintained by the investigator and made available for inspection.

The PI at each study site is also responsible for providing the IRB with reports of any reportable serious adverse drug reactions from any other study conducted with the investigational product per the IRB requirements. Medeor Therapeutics or its designee will provide this information to the PI. Progress reports will be provided to the IRB according to local regulations and guidelines.

#### Ethical conduct of the study

The study will be performed in accordance with ethical principles that have their origin in the Declaration of Helsinki and are consistent with ICH/GCP and applicable regulatory requirements.

#### Written informed consent

The investigator(s) at each center will ensure that the prospective study subjects are given full and adequate oral and written information about the nature, purpose, possible risk, and benefit of the study. Subjects will also be notified that they are free to discontinue from the study at any time. The subjects will be given the opportunity to ask questions and allowed time to consider the information provided.

### Data handling and record keeping

#### Case report form completion

The Sponsor or its designee will provide the clinical sites with access to an electronic case report form (eCRF) for each subject. The PI is responsible for ensuring the accuracy, completeness, and timeliness of the data reported in a subject’s eCRF. Source documentation supporting the eCRF data should indicate the subject’s participation in the study and should document the dates and details of study procedures, AEs, and subject status.

The investigator or designated representative should complete the eCRFs as soon as possible after information is collected. The PI must sign and date the eCRF to endorse the recorded data.

#### Inspection of records

The sponsor or its designee will be allowed to conduct site visits to the investigation facilities for the purpose of monitoring any aspect of the study. The investigator agrees to allow the monitor to inspect the subject charts and study source documents, and other records relative to study conduct.

#### Retention of records

A study investigator must maintain all documentation relating to the study for a period of 2 years after the last marketing application approval, or if not approved 2 years following the discontinuance of the test article for investigation or according to applicable regulatory requirements.

If the investigator withdraws from the responsibility of keeping the study records, custody must be transferred to a person willing to accept the responsibility. Medeor Therapeutics must be notified in writing if a custodial change occurs.

### Protocol deviations

A protocol deviation is any noncompliance with the clinical trial protocol, International Conference on Harmonization Good Clinical Practice (ICH GCP), or Manual of Procedures (MOP) requirements. The noncompliance may be either on the part of the participant, the investigator, or the study site staff. As a result of deviations, corrective actions are to be developed by the site and implemented promptly.

It is the responsibility of the site investigator to use continuous vigilance to identify and report deviations. Protocol deviations must be sent to the reviewing Institutional Review Board (IRB) per their policies. The site investigator is responsible for knowing and adhering to the reviewing IRB requirement.

## Discussion

The continued success of transplantation has been limited by adverse effects associated with immunosuppression. Immunosuppression is associated with increased risk for infections, malignancies, cardiovascular and metabolic disease (hypertension, hyperlipidemia, post-transplant diabetes mellitus), and allograft nephrotoxicity, and independently increases morbidity and mortality in transplant recipients [11]. In addition, chronic rejection, which can be immune or non-immune, can occur despite or because of use of immunosuppression, leading to progressive graft loss [12]. The generation of immune tolerance is the next frontier in transplantation to achieve continued durability of the graft without use of immunosuppression laden with adverse effects.

Various trials are now underway to assess the novel methodology of donor-derived CD34 + hematopoietic stem/progenitor cells and a specified dose of CD3 + T cells administered to recipients of kidney transplants post total lymphoid irradiation. While preliminary findings from these trials are extremely encouraging, long-term effects of this immune tolerance regimen are still largely unknown. Extension or “roll-over” studies are especially beneficial in the setting and can provide early evidence of outcomes, along signals of tolerability and safety issues [13].

The current study is unique given it combines multiple studies as a “master protocol,” a recent innovation in research methodology that can aid in evaluation of multiple therapies [14]. These studies have the advantage of utilizing existing infrastructure of trials being included in the master protocol and provide concurrent comparisons of therapies in different population groups [14].

The MDR-105-SAE is a long-term study that will provide long-term safety data in patients currently enrolled in various kidney transplant trials involving administration of immune tolerance regimes. It is imperative that these regimes be shown not only to be effective, but also be associated with long-term safety for the field of transplantation to take the next prodigious and essential step into the future.
